# Supplementary material for: Sinonasal Inverted Papilloma–Associated and De Novo Squamous Cell Carcinoma: A Tale of Two Cities or Not
Source: Cancers (Basel). 2022 Oct 24;14(21):5211. doi: 10.3390/cancers14215211 (PMC9658543; doi:10.3390/cancers14215211)
Supplement: Supplementary file 1 [file cancers-14-05211-s001.zip › Table S1.pdf]

Table S1. Cox Proportional Hazards Regression Analysis of Survival.

	DFS		DSS		OS	
Characteristics	HR (95%CI)	p-value	HR (95%CI)	p-value	HR (95%CI)	p-value
Age, years						
≤ 54	Ref		Ref		Ref	
> 54	1.118 (0.778-1.606)	0.546	1.394 (0.941-2.065)	0.098	1.590 (1.100-2.299)	0.014
Gender						
Male	Ref		Ref		Ref	
Female	0.583 (0.377-0.901)	0.015	0.756 (0.475-1.201)	0.236	0.849 (0.557-1.295)	0.448
Primary site						
Nasal cavity	Ref		Ref		Ref	
Maxillary sinus	1.140 (0.639-2.035)	0.657	1.168 (0.623-2.188)	0.628	1.258 (0.693-2.283)	0.450
Ethmoid sinus	1.029 (0.681-1.555)	0.894	0.976 (0.617-1.546)	0.919	1.079 (0.698-1.667)	0.733
Tumor etiology						
DN-SCC	Ref		Ref		Ref	
IP-SCC	1.207 (0.808-1.802)	0.359	1.256 (0.815-1.935)	0.301	1.116 (0.742-1.678)	0.599
Years of diagnosis						
2000-2009	Ref		Ref		Ref	
2010-2016	0.652 (0.437-0.973)	0.036	0.756 (0.484-1.178)	0.216	0.786 (0.521-1.188)	0.251
TNM stage (AJCC 8th)						
I-III	Ref		Ref		Ref	
IV	1.813 (1.149-2.862)	0.011	2.693 (1.547-4.688)	< 0.001	3.011 (1.784-5.081)	< 0.001
Treatment modalities						
Surgery + radiotherapy	Ref		Ref		Ref	
Single-modality therapy	2.572 (1.778-3.721)	< 0.001	2.896 (1.941-4.320)	< 0.001	2.658 (1.817-3.888)	< 0.001
Chemotherapy						
No	Ref		Ref		Ref	
Yes	1.365 (0.893-2.088)	0.151	1.412 (0.890-2.238)	0.143	1.314 (0.856-2.015)	0.212
