# Supplementary material for: Sinonasal Inverted Papilloma–Associated and De Novo Squamous Cell Carcinoma: A Tale of Two Cities or Not
Source: Cancers (Basel). 2022 Oct 24;14(21):5211. doi: 10.3390/cancers14215211 (PMC9658543; doi:10.3390/cancers14215211)
Supplement: Supplementary file 1 [file cancers-14-05211-s001.zip › Table S2.pdf]

Table S2. Comparison of baseline characteristics of IP-SCC vs DN-SCC for S+RT cohort.

Characteristics	Before PSM			After PSM		
	IP-SCC N = 53 (%)	DN-SCC N = 112 (%)	p-value	IP-SCC N = 40 (%)	DN-SCC N = 40 (%)	p-value
Age, years						
≤ 54	25 (47.2)	61 (54.5)	0.381	17 (42.5)	20 (50.0)	0.501
> 54	28 (52.8)	51 (45.5)		23 (57.5)	20 (50.0)	
Gender						
Male	42 (79.2)	81 (72.3)	0.340	30 (75.0)	30 (75.0)	1.000
Female	11 (20.8)	47 (27.7)		10 (25.0)	10 (25.0)	
Primary site						
Nasal cavity	23 (43.4)	25 (22.3)	0.014	14 (35.0)	12 (30.0)	0.900
Maxillary sinus	25 (47.2)	78 (69.6)		22 (55.0)	23 (57.5)	
Ethmoid sinus	5 (9.4)	9 (8.0)		4 (10.0)	5 (12.5)	
Years of diagnosis						
2000-2009	23 (43.4)	55 (49.1)	0.493	15 (37.5)	19 (47.5)	0.366
2010-2016	30 (56.6)	57 (50.9)		25 (62.5)	21 (52.5)	
T stage						
T1-2	2 (3.8)	10 (8.9)	0.491	1 (2.5)	1 (2.5)	0.936
T3	10 (18.9)	27 (24.1)		9 (22.5)	7 (17.5)	
T4a	25 (47.2)	48 (42.9)		19 (47.5)	21 (52.5)	
T4b	16 (30.2)	27 (24.1)		11 (27.5)	11 (27.5)	
N stage						
N0	48 (90.6)	87 (77.7)	0.045	35 (87.5)	35 (87.5)	1.000
N+	5 (9.4)	25 (22.3)		5 (12.5)	5 (12.5)	
TNM stage (AJCC 8th)						
I-II	2 (3.8)	7 (6.3)	0.549	1 (2.5)	1 (2.5)	0.883
III	9 (17.0)	25 (22.3)		8 (20.0)	6 (15.0)	
IV	42 (79.2)	80 (71.4)		31 (77.5)	33 (82.5)	
Chemotherapy						
No	45 (84.9)	72 (64.3)	0.006	33 (82.5)	34 (85.0)	0.762
Yes	8 (15.1)	40 (35.7)		7 (17.5)	6 (15.0)	
Surgical approaches						
Endoscopic surgery	18 (19.2)	14 (12.5)	0.256	5 (12.5)	10 (25.0)	0.152
Open surgery	42 (80.8)	98 (87.5)		35 (87.5)	30 (75.0)	
Margin status						
R0 resection	24 (46.2)	81 (73.6)	0.001	22 (55.0)	21 (52.5)	0.823
R1 / R2 resection	28 (53.8)	29 (26.4)		18 (45.0)	19 (47.5)	
Radiation technique						
Non-IMRT	20 (37.7)	51 (45.5)	0.345	14 (35.0)	17 (42.5)	0.491
IMRT	33 (62.3)	61 (54.5)		26 (65.0)	23 (57.5)	
Radiation dose, Gy						
≤ 66	25 (49.0)	64 (58.2)	0.277	19 (47.5)	22 (55.0)	0.502
> 66	26 (51.0)	46 (41.8)		21 (52.5)	18 (45.0)	
