# Supplementary material for: Sinonasal Inverted Papilloma–Associated and De Novo Squamous Cell Carcinoma: A Tale of Two Cities or Not
Source: Cancers (Basel). 2022 Oct 24;14(21):5211. doi: 10.3390/cancers14215211 (PMC9658543; doi:10.3390/cancers14215211)
Supplement: Supplementary file 1 [file cancers-14-05211-s001.zip › Table S3.pdf]

Table S3. Details of patients with regional relapse.

Characteristics	IP-SCC N = 7 (%)	DN-SCC N = 12 (%)
Primary site		
Nasal cavity	3 (42.9)	4 (33.3)
Maxillary sinus	3 (42.9)	6 (50.0)
Ethmoid sinus	1 (14.3)	2 (16.7)
Lymph node metastasis at presentation		
Yes	0 (0.0)	3 (25.0)
No	7 (100.0)	9 (75.0)
TNM stage (AJCC 8th)		
III	0 (0.0)	3 (25.0)
IVA	3 (42.9)	5 (41.7)
IVB	4 (57.1)	4 (33.3)
Treatment modalities		
Surgery plus radiotherapy	7 (100.0)	7 (58.3)
Radiotherapy	0 (0.0)	5 (41.7)
Neck irradiation		
Yes	4 (57.1)	8 (66.7)
No	3 (42.9)	4 (33.3)
Recurrence category		
In-field	3 (42.9)	5 (41.7)
Out-field	3 (42.9)	6 (50.0)
Unknown	1 (14.3)	1 (8.3)
Prior or concomitant local recurrence		
Prior local recurrence	3 (42.9)	2 (16.7)
Concomitant local recurrence	3 (42.9)	4 (33.3)
Without local recurrence	1 (14.3)	6 (50.0)
